# Robot-Assisted Radical Nephroureterectomy: A Safe and Effective Option for Upper Tract Urothelial Carcinoma, Especially for Novice Surgeons

**DOI:** 10.3390/cancers17091394

**Published:** 2025-04-22

**Authors:** Chia-Lun Chang, Chung-You Tsai, Pai-Yu Cheng, Wen-Jeng Wu, Yao-Chou Tsai

**Affiliations:** 1 Division of Urology, Department of Surgery, Taipei Tzu Chi Hospital, The Buddhist Medical Foundation, New Taipei 231405, Taiwan; mr.tony1990@gmail.com; 2 School of Medicine, Buddhist Tzu Chi University, Hualien 97004, Taiwan; 3 Division of Urology, Department of Surgery, Far Eastern Memorial Hospital, New Taipei 220216, Taiwan; pgtsai@gmail.com (C.-Y.T.); zack00693@gmail.com (P.-Y.C.); 4 Department of Electrical Engineering, Yuan-Ze University, Chung-Li 32003, Taiwan; 5 Department of Biomedical Engineering, National Taiwan University, Taipei 10617, Taiwan; 6 Department of Urology, Kaohsiung Medical University Hospital, Kaohsiung 80756, Taiwan; wejewu@kmu.edu.tw; 7 Department of Urology, School of Medicine, College of Medicine, Kaohsiung Medical University, Kaohsiung 80708, Taiwan; 8 Graduate Institute of Clinical Medicine, College of Medicine, Kaohsiung Medical University, Kaohsiung 80708, Taiwan

**Keywords:** robotic, laparoscopic, nephroureterectomy, upper urinary tract urothelial cancer

## Abstract

Background: Radical nephroureterectomy (RNU) is the standard treatment for upper tract urothelial carcinoma (UTUC). Minimally invasive techniques like robotic (RARNU) and laparoscopic (LRNU) RNU offer potential benefits over open surgery, but their comparative oncologic outcomes are debated. Methods: This retrospective, multicenter study analyzed 2037 Taiwanese patients undergoing RNU between 2010 and 2022. Missing data was addressed using multiple imputations. Overlap weighting was applied to balance patient characteristics between the RARNU and LRNU groups. Survival outcomes were compared using Kaplan-Meier analysis and Cox regression. Results: After excluding the missing data, 405 patients underwent RARNU, and 1262 underwent LRNU. After adjusting for baseline differences, both groups showed comparable rates of surgical complications, residual tumor, UTUC-related mortality, and disease recurrence. The median follow-up was similar (52.4 vs. 51.6 months, *p* = 0.91). Using Kaplan-Meier survival curve analysis, overall survival, cancer-specific survival, and disease-free survival were similar between the two groups. Conclusions: This study shows that RARNU for UTUC has comparable oncological and perioperative outcomes to pure LRNU. RARNU may be easier for surgeons to adopt, particularly those with limited laparoscopic experience.

## Simple Summary

This study compared robotic (RARNU) and laparoscopic (LRNU) surgery for treating upper tract urothelial carcinoma (UTUC). Researchers analyzed data from over 2037 Taiwanese patients and found that both approaches had similar outcomes in terms of complications, cancer recurrence, and survival rates. While both methods were effective, the study suggests that robotic surgery might be easier for surgeons to adopt, especially those with less experience in laparoscopic techniques.

## 1. Introduction

Upper tract urothelial carcinoma (UTUC) is rare, making up 5–10% of urothelial cancers in the West, but has a significantly higher incidence (20–30%) in Taiwan [1,2,3,4,5]. Unlike other regions, Taiwan also shows a higher prevalence of UTUC among women, suggesting a unique cancer behavior [6,7]. Radical nephroureterectomy (RNU) with bladder cuff excision is the standard treatment for UTUC [8]. Traditionally performed using an open approach, the rise of minimally invasive techniques has transformed the surgical landscape by offering advantages such as smaller incisions, better cosmetic results, reduced bleeding, and faster postoperative recovery [9]. The use of laparoscopic and robot-assisted RNU increased from 36% to 54% between 2004 and 2013 [10]. Several studies have shown that LRNU and RARNU offer reduced perioperative morbidity and comparable oncological control compared to the open approach [11,12,13]. However, the potential for incomplete tumor resection, increased tumor recurrence, and long-term oncological outcomes associated with minimally invasive approaches—including LRNU and RARNU—remain debated, with mixed results in the literature [14,15,16,17]. Moreover, many previous studies have analyzed small cohorts commonly enrolled using the hand-assisted approach and often failed to assess oncological outcomes comprehensively [10,18,19]. In contrast to the hand-assisted laparoscopic approach, pure LRNU presents a steeper learning curve and poses significant challenges, particularly in managing the bladder cuff. These conflicting findings underscore the need for more rigorous and comprehensive analyses to better understand the true benefits and limitations of pure LRNU and RARNU in the treatment of UTUC. This study aims to comprehensively compare the safety, efficacy, and impact of pure LRNU and RARNU for UTUC during the learning curve in a multi-institutional Taiwanese cohort. The findings will inform clinical decision-making and optimize surgical management strategies for patients with UTUC.

## 2. Materials and Methods

### 2.1. Study Population

**Figure 1:**
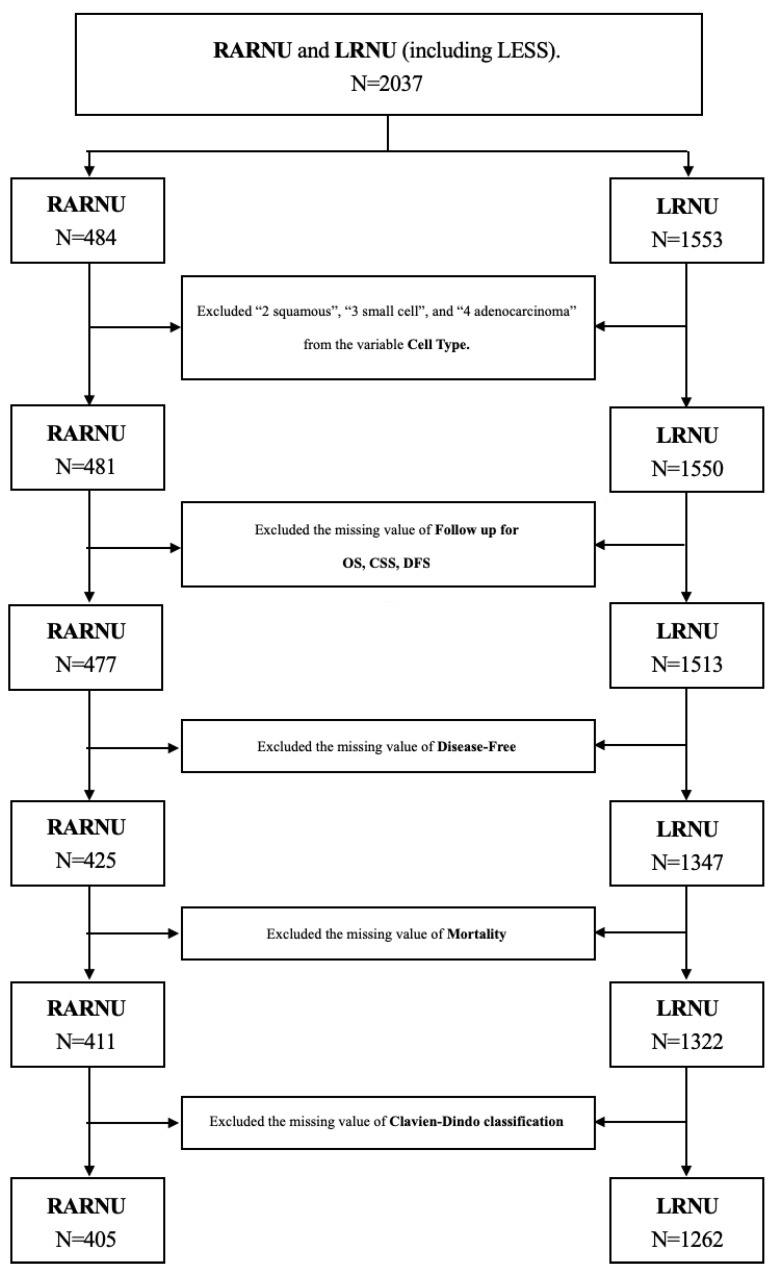
Study flow diagram.

The Taiwan UTUC Collaboration Group Database compiled clinical and pathological data of UTUC patients from 21 hospitals across Taiwan. This study retrospectively analyzed data from patients who underwent RARNU or LRNU, including laparoendoscopic single-site (LESS) RNU, between 2010 and 2022. Exclusion criteria included clinical evidence of metastatic disease at the time of surgery, a prior history of cystectomy for bladder cancer, hand-assisted LRNU, and variant UTUC subtypes. Out of 2037 patients assessed for eligibility, 1667 were included in the final analysis (Figure 1 flow diagram). All the participants underwent RNU with bladder cuff excision. Lymph node dissection was performed only for those with suspected lymph node involvement, with the extent determined by the surgeon. Decisions regarding neoadjuvant or adjuvant chemotherapy were based on the patient’s clinical staging and overall condition, as decided by the treating physician and patient. This retrospective analysis was approved by the Institutional Review Board of Taipei Tzu Chi Hospital (No. 06-X34-105).

### 2.2. Definitions and Endpoints

Pathological staging was based on the 2010 TNM (tumor, lymph node, metastasis) system, and tumor grading followed the 2004 WHO/International Society of Urologic Pathology classification. Postoperative complications were classified using the Clavien–Dindo system. Recurrence was confirmed by cross-sectional imaging and/or pathological examination, including local recurrence at the tumor bed, lymph nodes, or distant sites. Disease-free survival (DFS) was defined as the time from surgery to the first recurrence, while overall survival (OS) was defined as the time from surgery to death from any cause. The cause of death was determined mainly from death certificates, with medical records reviewed when unclear. The study’s endpoints were the comparison of oncological outcomes and safety between RARNU and LRNU, specifically focusing on overall survival (OS), cancer-specific survival (CSS), and disease-free survival (DFS).

### 2.3. Follow-Up Protocols

Patients were generally evaluated every 3–6 months through a medical history review, physical examination, urine cytology, renal ultrasound, and cystoscopy. Abdominal computed tomography (CT) or magnetic resonance imaging (MRI) was performed every 6–12 months to monitor for recurrence. If clinically indicated, chest CT or a bone scan was conducted to check for distant metastasis.

### 2.4. Statistical Analysis

Differences between the RARNU and LRNU (including LESS) groups were assessed using standardized mean differences for both categorical and continuous variables. Approximately 4% of the data had missing values across some variables. We addressed these missing values using multiple imputations by chained equations (MICE), a commonly applied method in literature. To assess the performance of MICE in our context, we simulated a dataset with missing values similar to those observed. We first created a complete dataset by removing all rows containing missing values from the original data, which consisted of 1028 patients and 30 variables. From this complete dataset, we randomly deleted 5000 values from the variables that originally had missing data to construct a testing dataset with simulated missingness. The accuracy of the MICE imputations, averaged over 30 trials, was 0.72 [20] reported that acceptable accuracy in machine learning models typically ranges from 0.7 to 0.9; our results indicate that MICE performed adequately in this scenario.

To balance the two groups, we applied overlap weighting to adjust for baseline differences. Overlap weighting is a propensity score (PS) matching method that has several advantages over other PS matching methods, including: (1) It creates an exact balance on the mean of every measured covariate when the PS is estimated by logistic regression. This is particularly important for reducing bias, as it ensures that the treatment and control groups are comparable in all the measured confounders. (2) It can be used even when there are no overlaps between the treatment and control groups on the PS distribution, which is common in a retrospective setting. (3) It does not require trimming of case numbers. (4) Overlap weighting has been shown to minimize the variance of the weighted estimator of the treatment effect among all the balancing weights, including inverse probability of treatment weighting (IPTW). This means that overlap weighting is more efficient than other PS matching methods in terms of precision [21,22]. After the overlap weighting, the love plot revealed a balanced standardized mean difference across LRNU vs. RARNU groups among all variables with the overlap technique (Supplementary Figure S1: Love Plot). In addition, the performance of overlap weighting was clearly better than the inverse probability treatment weighting technique.

For the survival analyses, we prioritized variable selection using the Akaike Information Criterion (AIC) and Lasso regression to identify the most relevant variables. The Kaplan-Meier estimator, combined with Cox regression modeling, was then used to generate and visualize the overall, cancer-specific, and disease-free survival curves.

## 3. Results

### 3.1. Baseline Characteristics

A total of 2037 patients who underwent minimally invasive radical nephroureterectomy (RNU) across 21 centers in Taiwan were analyzed. After excluding missing values and imputing missing data using multiple imputations by chained equations (MICE), 405 patients had robot-assisted RNU (RARNU) and 1262 had pure laparoscopic RNU (LRNU) for final analysis (Table 1).

Before overlap weighting, the baseline characteristics were comparable among most variables, except for surgical margin status, bladder cuff resection methods, and postoperative intravesical chemotherapy status. The RARNU group was associated with a higher rate of high-grade histology and positive surgical margins (5.2% vs. 2.9%, *p* = 0.037). Additionally, nearly 75% of bladder cuffs in the RARNU group were managed endoscopically, compared to only 23% in the LRNU group. Intravesical chemotherapy after RNU was administered to a limited number of patients in both groups (17.3% vs. 4.9%, *p* < 0.001).

### 3.2. Surgical Outcomes

After overlap weighting, the standardized mean differences were well-balanced across groups for all baseline characteristics (Supplementary Figure S1). The mean age in the RARNU group was 69.25 ± 10.26 years and 68.15 ± 10.51 years in the LRNU group (Table 1).

**Figure 2:**
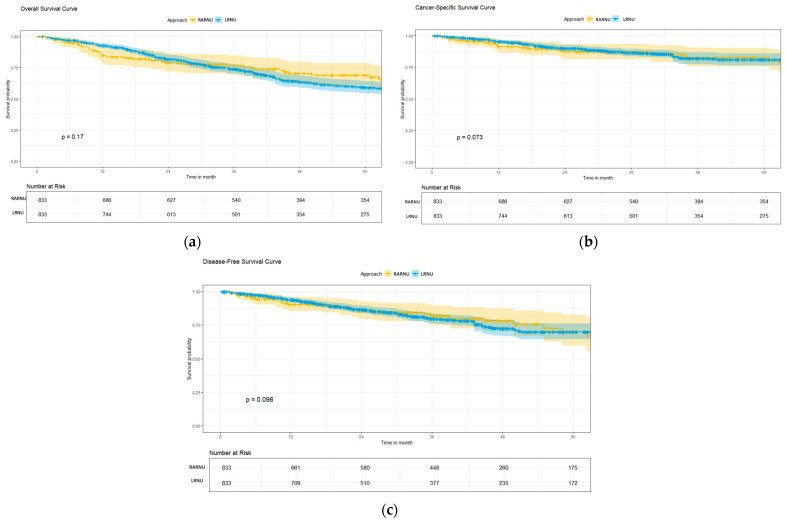
Survival analysis of UTUC patients with robot-assisted or laparoscopic radical nephroureterectomy using Kaplan-Meier method (**a**) 5-year overall survival, (**b**) 5-year cancer-specific survival, and (**c**) 5-year disease-free survival.

The outcome variables derived after overlap weighting revealed comparable rates of Clavien-Dindo surgical complications, rate of residual bladder cuff, UTUC-related mortality, and disease recurrence between the two groups, with comparable median follow-up periods (52.4 ± 33.4 vs. 51.6 ± 37.8 months, *p* = 0.91) (Table 2). The Kaplan–Meier survival curve analysis demonstrated similar OS, CSS, and DFS between the two groups (Figure 2).

### 3.3. Survival Prediction Model

Multivariate survival analysis revealed comparable OS, CSS, and DFS between the two groups (Table 3). Independent risk factors for OS included higher ECOG, higher age, multiplicity, advanced pathological stage, and positive surgical margin. Independent risk factors for CSS included higher age, advanced pathological stage, multiplicity, tumor > 2 cm, LVI, and positive surgical margin. Independent risk factors for DFS included higher age, multiplicity, advanced pathological stage, concurrent bladder urothelial carcinoma (UC), lymphovascular invasion (LVI), positive surgical margin, and hydronephrosis.

### 3.4. Surgeon Experience

In our sub-analysis, the median surgical loads per doctor were three for the RARNU group and four for the LRNU group. The average surgical loads per doctor were 8.21 for the RARNU group and 8.52 for the laparoscopic surgery LRNU group. Additionally, according to the case load plot, nearly half (25/56) of robotic surgeons had no or minimal experience (less than 10 cases in LRNU). (Supplementary Figure S2).

## 4. Discussion

Robot-assisted radical nephroureterectomy (RARNU) offers several advantages over open surgery, including smaller incisions, improved cosmetic outcomes, reduced blood loss, and faster postoperative recovery [23]. While laparoscopic radical nephroureterectomy (LRNU) shares these benefits, its steep learning curve has hindered widespread adoption of minimally invasive approaches in renal surgery [24]. The introduction of robotic platforms has facilitated the transition from open to minimally invasive surgery, making endoscopic urological procedures more accessible to patients and surgeons alike [25,26]. Our multicenter study represents one of the largest cohort analyses comparing surgical and oncological outcomes for upper tract urothelial carcinoma (UTUC) patients undergoing robotic and pure laparoscopic RNU. We found that RARNU was associated with comparable hospital stays, surgical complications, and survival outcomes compared to pure LRNU. These findings suggest that RARNU is a safe and effective surgical option for UTUC and may provide a more accessible pathway for open surgeons to adopt minimally invasive techniques.

Robotic surgical systems have significantly advanced minimally invasive surgery, empowering surgeons to perform complex procedures endoscopically with greater precision, dexterity, and control [9,27]. Radical prostatectomy (RP), one of the most intricate endoscopic procedures in urology, exemplifies this revolution. In the United States, a national survey between 1998 and 2011 demonstrated a rapid expansion of minimally invasive RP, driven by the widespread adoption of da Vinci Surgical Systems [28]. Notably, during this period, laparoscopic RP declined to less than 10% of all RP procedures, highlighting the versatility and ease of adaptation of the robotic platform for open-incision surgeons. Although the da Vinci Surgical System received FDA approval in Taiwan in 2004, the first case of RARNU in the country was performed in 2011, marking the start of the learning curve for RARNU in this cohort study. According to the case load plot, approximately half of the robotic surgeons had experience with fewer than 10 LRNU cases. Although a significant proportion of surgeons in the RARNU group had no experience or were in their early learning curve, both groups had comparable surgical and oncological outcomes. Therefore, the RARNU approach is a versatile and user-friendly procedure, particularly for young and novice surgeons, with similar surgical outcomes to its pure laparoscopic counterpart.

Bladder cuff resection is one of the critical components of radical nephroureterectomy, particularly for tumors located in the lower ureter or bladder cuff. Pure endoscopic management of the bladder cuff offers several advantages, including smaller incisions, reduced blood loss, faster recovery, and potentially decreased risks of open wound-related complications [29]. However, laparoscopic techniques, while minimally invasive, have been associated with a higher incidence of positive surgical margins and local recurrence in some smaller retrospective studies [30,31]. Additionally, the technical challenges of intracorporeal suturing can limit the widespread adoption of laparoscopic approaches, especially for open-incision surgeons. In contrast, RARNU provides a more versatile and accessible platform for bladder cuff resection. The 3D magnified view and enhanced dexterity afforded by robotic technology allow for precise dissection and suturing, even in complex cases. Our multicenter study demonstrated that RARNU facilitated endoscopic bladder cuff resection in over 70% of cases, compared to only 25% in the laparoscopic group. Though most bladder cuffs were managed endoscopically in the RARNU group during the learning curve, the RARNU group actually had a similar bladder cuff residual rate (21.7 vs. 21.4%, *p* = 0.95) and bladder recurrence rate (26.0 vs. 26.1%, *p* = 0.98) as the LRNU group (Table 2). Moreover, the current study and other large-scale studies have all shown that RARNU does not increase the risk of positive surgical margins or disease recurrence [11,17,32]. Hence, robot-assisted surgery offers a safe, effective, and more versatile endoscopic approach to bladder cuff resection during radical nephroureterectomy.

The results in Table 3 also demonstrated that there was no statistically significant difference between the RARNU and LRNU groups in terms of OS, CSS, or DFS in this multivariable model. This finding is central to our comparison, suggesting that after accounting for other factors, the surgical approach itself does not independently impact long-term oncological outcomes within our study cohort. The absence of a significant difference between the groups in this analysis supports our findings regarding the comparable long-term effectiveness of both techniques. By combining the benefits of minimally invasive surgery with enhanced surgical precision, the robotic platform provides a valuable tool to optimize patient outcomes and minimize complications in bladder cuff resection.

Hand-assisted laparoscopic nephroureterectomy (HALRNU) can help overcome the learning curve associated with the pure laparoscopic approach for upper tract urothelial carcinoma (UTUC). Historical comparative cohorts for robotic versus laparoscopic RNU commonly enrolled cases using the hand-assisted approach for LRNU [33]. While HALRNU might shorten operative times due to hand assistance for blunt dissection and tactile feedback, this advantage does not significantly impact overall survival, cancer-specific mortality, or extra-vesical recurrence rates. However, HALRNU may increase the risk of intravesical recurrence and a higher rate of disease recurrence following RNU [33,34]. This could be due to the increased possibility of genitourinary tract manipulation during hand-assisted surgery, thus increasing the chance of tumor dissemination to the urinary bladder or lymphatic tracts, leading to disease recurrence. In our cohort analysis, RARNU not only performed as well as pure LRNU but also maintained the treatment quality of pure LRNU during the learning phase. Therefore, RARNU should be recommended as the treatment of choice for novice surgeons treating UTUC.

While RARNU offers several advantages over LRNU, such as a more user-friendly surgical platform for novice surgeons, improved surgeon ergonomics, and potentially easier endoscopic bladder cuff management, it is not without its drawbacks. Firstly, RARNU typically requires a longer operative time compared to LRNU [35]. Secondly, the use of robotic systems often results in higher surgical costs. These increased costs stem from the significant expense of acquiring and maintaining the robotic equipment [35,36]. Finally, not all medical centers have access to robotic surgical systems, which can limit the availability of RARNU for patients [37]. Therefore, the choice between RARNU and LRNU should be made on a case-by-case basis, through careful discussion between the patient and their urologist. This discussion should consider several factors, including surgeon proficiency with both techniques, availability of robotic platforms, patient affordability, and hospital resources.

Our study should be addressed with some limitations. Being a retrospective study, it inherently carries the potential for bias and confounding variables. The multicenter nature of the study, involving different surgeons, adds to the heterogeneous nature of the data. Different surgeons with varying levels of experience and differing surgical methods may have influenced the results. However, the study was strengthened by its real-world data analysis with a large sample size and comprehensive correction for confounding covariates.

## 5. Conclusions

This study demonstrates that RARNU is a safe and effective surgical option for the treatment of UTUC. Our findings show that RARNU is associated with comparable oncological and perioperative outcomes to pure laparoscopic radical nephroureterectomy, including similar rates of complications, recurrence, and survival. Importantly, RARNU may provide a more user-friendly and accessible pathway for surgeons, particularly those with limited laparoscopic experience, to adopt minimally invasive techniques.

## Figures and Tables

**Table 1:** Comparison of baseline patient characteristics, tumor characteristics, pathological findings, postoperative complications, and treatment outcomes in UTUC patients undergoing robot-assisted or laparoscopic radical nephroureterectomy after multiple imputation with chained equations.

		RARNU	LRNU	*p*	SMD
N		405	1262		
	NU	394	1257		
	Segmental	11	5		
ECOG (%)	0	211 (52.1)	631 (50.0)	0.217	0.153
	1	172 (42.5)	521 (41.3)		
	2	20 (4.9)	89 (7.1)		
	3	2 (0.5)	16 (1.3)		
	4	0 (0.0)	5 (0.4)		
Sex (%)	Male	172 (42.5)	520 (41.2)	0.695	0.026
	Female	233 (57.5)	742 (58.8)		
Age (mean (SD))		69.25 (10.26)	68.15 (10.51)	0.066 *	0.106
BMI (%)	Normal	241 (59.5)	776 (61.5)	0.513	0.041
	Overweight	164 (40.5)	486 (38.5)		
Cell Type (%)	Urothelial	346 (85.4)	1124 (89.1)	0.126	0.112
	UC with variants	55 (13.6)	131 (10.4)		
	Others	4 (1.0)	7 (0.6)		
Side (%)	Left	209 (51.6)	643 (51.0)	0.948	0.019
	Right	189 (46.7)	599 (47.5)		
	Both	7 (1.7)	20 (1.6)		
Location (%)	Non-Visible	1 (0.2)	1 (0.1)	0.645	0.086
	Renal Pelvis	163 (40.2)	546 (43.3)		
	Ureter	142 (35.1)	402 (31.9)		
	Bladder Cuff	1 (0.2)	2 (0.2)		
	Multiple	98 (24.2)	311 (24.6)		
Multiple (%)	No	262 (64.7)	825 (65.4)	0.849	0.014
	Yes	143 (35.3)	437 (34.6)		
Size (%)	<2 cm	131 (32.3)	416 (33.0)	0.865	0.013
	≥2 cm	274 (67.7)	846 (67.0)		
Pathological Stage (%)	Stage 0a/0is	64 (15.8)	204 (16.2)	0.167	0.14
	Stage I	104 (25.7)	359 (28.4)		
	Stage II	70 (17.3)	238 (18.9)		
	Stage III	125 (30.9)	375 (29.7)		
	Stage IV	42 (10.4)	86 (6.8)		
Grade (%)	Low Grade	43 (10.6)	122 (9.7)	0.001 **	0.237
	High Grade	351 (86.7)	1039 (82.3)		
	Not Available	11 (2.7)	101 (8.0)		
Bladder Cancer (%)	No	316 (78.0)	936 (74.2)	0.268	0.094
	Previous History of Bladder UC	24 (5.9)	96 (7.6)		
	Concurrent Bladder UC	65 (16.0)	230 (18.2)		
CIS (%)	No	280 (69.1)	919 (72.8)	0.17	0.081
	Yes	125 (30.9)	343 (27.2)		
LVI (%)	No	326 (80.5)	1051 (83.3)	0.226	0.072
	Yes	79 (19.5)	211 (16.7)		
Surgical Margin (%)	Free	384 (94.8)	1226 (97.1)	0.037 *	0.119
	Positive	21 (5.2)	36 (2.9)		
Pre-operation Hydronephrosis (%)	No	230 (56.8)	660 (52.3)	0.129	0.09
	Yes	175 (43.2)	602 (47.7)		
Tumor Necrosis (%)	No	352 (86.9)	1079 (85.5)	0.53	0.041
	Yes	53 (13.1)	183 (14.5)		
Chemotherapy Type (%)	No	275 (67.9)	907 (71.9)	0.062 *	0.128
	Peri-OP Adjuvant	99 (24.4)	295 (23.4)		
	Salvage/Palliative	31 (7.7)	60 (4.8)		
Bladder Cuff Resection (%)	Not Perform BCR	11 (2.7)	42 (3.3)	<0.001 ***	2.402
	Open Incision	81 (20.0)	879 (69.7)		
	Residual Bladder Cuff (%)	14 (17.3)	166 (18.9)		
	Transurethral Incision	9 (2.2)	52 (4.1)		
	Laparoscopy	7 (1.7)	284 (22.5)		
	Residual Bladder Cuff (%)		46 (16.2)		
	Robot-Assisted	297 (73.3)	5 (0.4)		
	Residual Bladder Cuff (%)	42 (14.1)			
Post-Operation Intravesical C/T Instillation (%)	No	335 (82.7)	1200 (95.1)	<0.001 ***	0.402
	Intravesical Therapy	70 (17.3)	62 (4.9)		

RARNU: robot-assisted radical nephroureterectomy, LRNU: laparoscopic radical nephroureterectomy, SMD: standardized mean difference, NU: nephroureterectomy, UC: urothelial carcinoma, CIS: carcinoma in situ, LVI: lymphovascular invasion, C/T: chemotherapy, *p*: <0.1 *, <0.01 **, <0.001 ***.

**Table 2:** Outcome variable comparisons between robot-assisted or laparoscopic radical nephroureterectomy groups before and after overlap weighting.

		Overlap				Unweighted			
		RARNU	LRNU	*p*	SMD	RARNU	LRNU	*p*	SMD
Clavien-Dindo Classification (%)	No Complication	468.6 (56.3)	541.5 (65.0)	0.122	0.245	259 (64.0)	861 (68.2)	0.079 *	0.180
	Grade I	154.7 (18.6)	105.3 (12.6)			66 (16.3)	148 (11.7)		
	Grade II	194.4 (23.3)	160.5 (19.3)			71 (17.5)	205 (16.2)		
	Grade III	14.7 (1.8)	18.2 (2.2)			6 (1.5)	31 (2.4)		
	Grade IV	0.3 (0.0)	7.3 (0.9)			2 (0.5)	16 (1.3)		
	Grade V	0.2 (0.0)	0.1 (0.0)			1 (0.2)	1 (0.1)		
Residual Bladder Cuff (%)	No	656.1 (78.8)	652.7 (78.4)	0.921	0.010	339 (83.7)	1020 (80.8)	0.22	0.075
	Yes	176.9 (21.2)	180.3 (21.6)			66 (16.3)	242 (19.2)		
Overall Mortality (%)	No	575.5 (69.1)	487.2 (58.5)	0.034 *	0.222	285 (70.4)	747 (59.2)	<0.001 ***	0.236
(Within 5 years)	Yes	257.5 (30.9)	345.8 (41.5)			120 (29.6)	515 (40.8)		
UTUC Mortality (%)	No	687.8 (82.6)	696.1 (83.6)	0.793	0.027	344 (84.9)	1102 (87.3)	0.252	0.069
(Within 5 years)	Yes	145.2 (17.4)	136.9 (16.4)			61 (15.1)	160 (12.7)		
Disease-Free (%)	No	176.1 (21.1)	183.4 (22.0)	0.834	0.021	98 (24.2)	244 (19.3)	0.042 *	0.118
	Yes	656.9 (78.9)	649.6 (78.0)			307 (75.8)	1018 (80.7)		
Bladder Recurrence (%)	No	616.4 (74.0)	615.5 (73.9)	0.981	0.002	308 (76.0)	921 (73.0)	0.247	0.07
	Yes	216.6 (26.0)	217.5 (26.1)			97 (24.0)	341 (27.0)		
Follow-Up OS/CSS (Mean(SD))		52.03 (33.43)	51.65 (37.79)	0.913	0.011	44.45 (30.64)	52.51 (38.88)	<0.001 ***	0.23

RARNU: robot-assisted radical nephroureterectomy, LRNU: laparoscopic radical nephroureterectomy, SMD: standardized mean difference, UTUC: upper tract urothelial carcinoma, OS: overall survival, CSS: cancer-specific survival, *p*: <0.1 *, <0.001 ***.

**Table 3:** Multivariable Cox regression analyses assessing the association of each variable with overall survival, cancer-specific survival, and disease-free survival in UTUC patients undergoing robot-assisted or laparoscopic radical nephroureterectomy.

		OS		CSS		DFS	
		HR (95%CI)	*p*	HR (95%CI)	*p*	HR (95%CI)	*p*
Approach	RARNU	1		1		1	
	LRNU	1.21 (0.85, 1.71)	0.298	0.80 (0.49, 1.30)	0.362	0.80 (0.63, 1.02)	0.071 *
ECOG	0	1				1	
	1	1.22 (1.03, 1.46)	0.024 *			1.33 (1.06, 1.68)	0.015 *
	2	1.82 (1.38, 2.41)	<0.001 ***			1.29 (0.84, 1.99)	0.25
	3	2.02 (1.20, 3.38)	0.008 **			1.74 (0.54, 5.57)	0.352
	4	1.53 (0.56, 4.19)	0.406			2.06 (0.28,14.92)	0.476
Sex	Male	1		1			
	Female	0.80 (0.68, 0.95)	0.009 **	0.70 (0.53, 0.92)	0.010 *		
Age	Mean	1.03 (1.02, 1.04)	<0.001 ***	1.04 (1.02, 1.05)	<0.001 ***	1.03 (1.01, 1.04)	<0.001 ***
BMI	Normal	1					
	Overweight	0.79 (0.67, 0.94)	0.007 **				
Multiple	No	1		1		1	
	Yes	1.22 (1.04, 1.45)	0.017 *	1.65 (1.26, 2.16)	<0.001 ***	1.26 (1.00, 1.58)	0.049 *
Size	<2 cm			1		1	
	≥2 cm			1.50 (1.02, 2.19)	0.038 *	1.28 (0.96, 1.71)	0.087 *
Pathological Stage	Stage 0a/0is	1		1		1	
	Stage I	1.34 (0.98, 1.83)	0.067 *	2.32 (0.96, 5.63)	0.062 *	1.31 (0.76, 2.25)	0.327
	Stage II	1.35 (0.96, 1.90)	0.088 *	3.58 (1.48, 8.66)	0.005 **	1.84 (1.06, 3.17)	0.030 *
	Stage III	2.04 (1.47, 2.84)	<0.001 ***	5.25 (2.22, 12.42)	<0.001 ***	3.32 (1.97, 5.60)	<0.001 ***
	Stage IV	5.06 (3.35, 7.64)	<0.001 ***	13.81 (5.55, 34.36)	<0.001 ***	8.31 (4.62,14.93)	<0.001 ***
Grade	Low Grade	1					
	High Grade	1.58 (1.08, 2.32)	0.019 *				
	Not Available	3.13 (2.03, 4.83)	<0.001 ***				
Bladder Cancer	No	1				1	
	Previous History of Bladder UC	0.99 (0.72, 1.37)	0.957			1.36 (0.85, 2.17)	0.204
	Concurrent Bladder UC	1.23 (1.01, 1.51)	0.043 *			1.36 (1.02, 1.80)	0.034 *
CIS	No	1				1	
	Yes	0.81 (0.67, 0.98)	0.026 *			0.75 (0.59, 0.96)	0.023 *
LVI	No	1		1		1	
	Yes	1.19 (0.97, 1.47)	0.096 *	1.42 (1.04, 1.93)	0.025 *	1.37 (1.06, 1.77)	0.015 *
Surgical Margin	Free	1		1		1	
	Positive	1.64 (1.14, 2.35)	0.007 **	1.67 (1.05, 2.65)	0.029 *	1.70 (1.16, 2.47)	0.006 **
Pre-operation Hydronephrosis	No					1	
	Yes					0.78 (0.62, 0.99)	0.039 *
Bladder Cuff Resection	Not Perform BCR	1		1			
	Open Incision	0.52 (0.34, 0.79)	0.002 **	0.57 (0.28, 1.19)	0.137		
	Transurethral Incision	0.31 (0.17, 0.56)	<0.001 ***	0.53 (0.21, 1.34)	0.181		
	Laparoscopy	0.39 (0.25, 0.61)	<0.001 ***	0.50 (0.23, 1.07)	0.075 *		
	Robot-Assisted	0.48 (0.28, 0.83)	0.009 **	0.44 (0.19, 1.03)	0.058 *		
Residual Bladder Cuff	No	1		1		1	
	Yes	0.54 (0.42, 0.70)	<0.001 ***	0.55 (0.36, 0.82)	0.004 **	0.76 (0.58, 1.01)	0.058 *

RARNU: robot-assisted radical nephroureterectomy, LRNU: laparoscopic radical nephroureterectomy, HR: hazard ratio, CI: confidence interval, OS: overall survival, CSS: cancer-specific survival, DFS: disease-free survival, UC: urothelial carcinoma, CIS: carcinoma in situ, LVI: lymphovascular invasion, *p*: <0.1 *, <0.01 **, <0.001 ***.

## Data Availability

The datasets analyzed during the current study are available from the corresponding author on reasonable request.

## Supplementary Materials

The following supporting information can be downloaded at: https://www.mdpi.com/article/10.3390/cancers17091394/s1, Figure S1: Love plot of the absolute standardized difference for each covariate in the original and weighted data: IPTW: inverse probability treatment weighting; overlap: overlap weighting; Figure S2: Case load plot illustrating the number of robotic-assisted and laparoscopic radical nephroureterectomy procedures performed by each surgeon.
