# Supplementary material for: Robot-Assisted Radical Nephroureterectomy: A Safe and Effective Option for Upper Tract Urothelial Carcinoma, Especially for Novice Surgeons
Source: Cancers (Basel). 2025 Apr 22;17(9):1394. doi: 10.3390/cancers17091394 (PMC12071112; doi:10.3390/cancers17091394)
Supplement: Supplementary file 1 [file cancers-17-01394-s001.zip › cancers-3513614-supplementary.pdf]

Supplementary Materials:

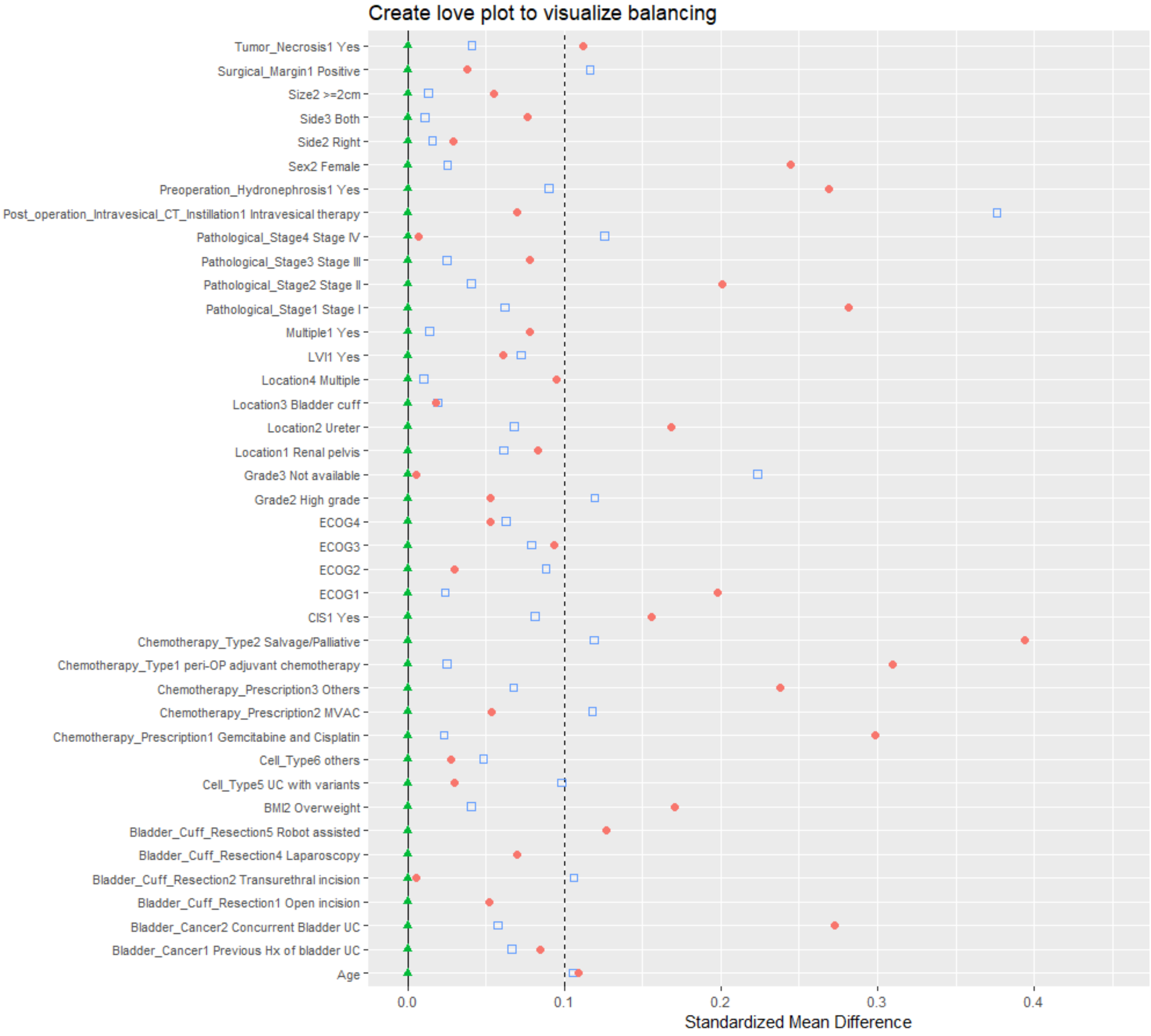

Supplementary Figure (1)

Supplementary Fig 1. Love plot of the absolute standardized difference for each covariate in the original and weighted data. IPTW: inverse probability treatment weighting; overlap: overlap weighting

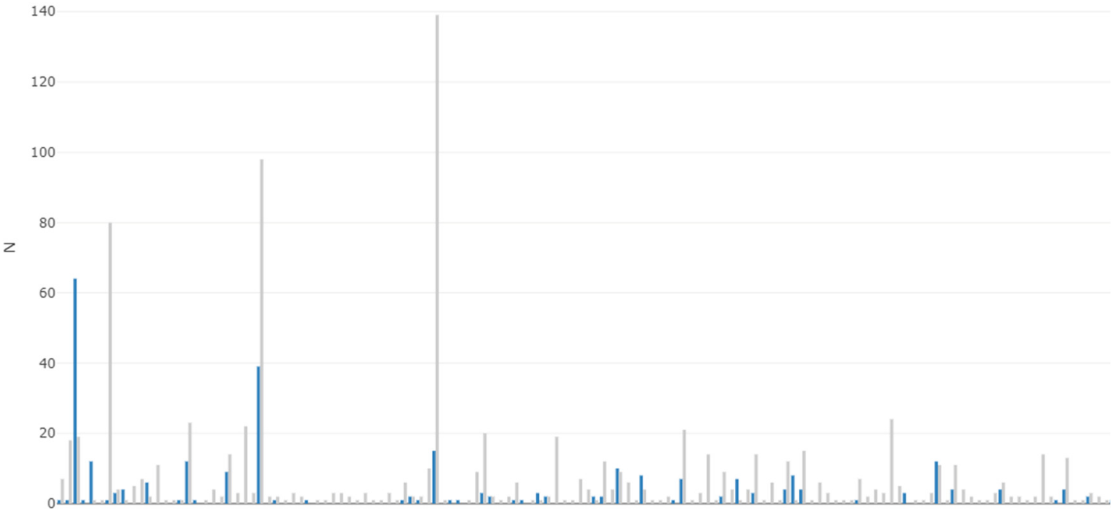

Supplementary Figure (2)

Supplementary Fig 2 Case load plot illustrating the number of robotic-assisted and laparoscopic radical nephroureterectomy procedures performed by each surgeon
